# Elevated inflammatory responses and targeted therapeutic intervention in a preclinical mouse model of ataxia-telangiectasia lung disease

**DOI:** 10.1038/s41598-021-83531-3

**Published:** 2021-02-19

**Authors:** Rudel A. Saunders, Thomas F. Michniacki, Courtney Hames, Hilary A. Moale, Carol Wilke, Molly E. Kuo, Johnathan Nguyen, Andrea J. Hartlerode, Bethany B. Moore, JoAnn M. Sekiguchi

**Affiliations:** 1 Department of Internal Medicine, University of Michigan, 109 Zina Pitcher Place, 2063 BSRB, Box 2200, Ann Arbor, MI 48109 USA; 2 Department of Pediatric Hematology/Oncology, University of Michigan, Ann Arbor, MI USA; 3 Department of Microbiology and Immunology, University of Michigan, Ann Arbor, MI USA; 4 Department of Human Genetics, University of Michigan, Ann Arbor, MI USA; 5 Department of Pathology, University of Michigan, Ann Arbor, MI USA

**Keywords:** Immunological disorders, Inflammation, Experimental models of disease, DNA damage and repair, Preclinical research

## Abstract

Ataxia-telangiectasia (A-T) is an autosomal recessive, multisystem disorder characterized by cerebellar degeneration, cancer predisposition, and immune system defects. A major cause of mortality in A-T patients is severe pulmonary disease; however, the underlying causes of the lung complications are poorly understood, and there are currently no curative therapeutic interventions. In this study, we examined the lung phenotypes caused by ATM-deficient immune cells using a mouse model of A-T pulmonary disease. In response to acute lung injury, ATM-deficiency causes decreased survival, reduced blood oxygen saturation, elevated neutrophil recruitment, exaggerated and prolonged inflammatory responses and excessive lung injury compared to controls. We found that *ATM* null bone marrow adoptively transferred to WT recipients induces similar phenotypes that culminate in impaired lung function. Moreover, we demonstrated that activated ATM-deficient macrophages exhibit significantly elevated production of harmful reactive oxygen and nitrogen species and pro-inflammatory cytokines. These findings indicate that ATM-deficient immune cells play major roles in causing the lung pathologies in A-T. Based on these results, we examined the impact of inhibiting the aberrant inflammatory responses caused by ATM-deficiency with reparixin, a CXCR1/CXCR2 chemokine receptor antagonist. We demonstrated that reparixin treatment reduces neutrophil recruitment, edema and tissue damage in *ATM* mutant lungs. Thus, our findings indicate that targeted inhibition of CXCR1/CXCR2 attenuates pulmonary phenotypes caused by ATM-deficiency and suggest that this treatment approach represents a viable therapeutic strategy for A-T lung disease.

## Introduction

Ataxia-telangiectasia (A-T) is an autosomal recessive, genomic instability disorder that is caused by mutations in the ataxia-telangiectasia mutated gene, *ATM,* leading to the production of a dysfunctional ATM protein. A-T patients exhibit pleiotropic clinical phenotypes, including cerebellar degeneration, oculocutaneous telangiectasia, immunodeficiency, and cancer predisposition^1–4^. One of the leading causes of morbidity and mortality in A-T patients is severe respiratory disease, which has been estimated to be the cause of death in up to one third of A-T patients^5^. Pulmonary manifestations in A-T result from episodes of acute insults, such as recurrent sinopulmonary infections and aspirations, leading to progressive bronchiectasis and chronic interstitial lung disease^6^. However, despite its importance as a cause of mortality in affected individuals, many questions regarding the molecular and cellular mechanisms underlying A-T lung disease remain unanswered, and there are currently no effective therapies to cure or prevent progression of this fatal outcome.

The ATM protein is a large serine-threonine protein kinase that is activated by chromosomal DNA double-strand breaks (DSBs) or direct oxidation in a DNA damage-independent manner^7^. Upon activation, ATM controls a broad range of cellular processes that function to maintain genome stability and oxidative homeostasis. In response to DNA DSBs, ATM phosphorylates a large number of proteins that effect DNA repair, cell cycle checkpoint activation or apoptosis^8,9^**.** In oxidative defense, ATM regulates oxidative-stress signaling pathways, glutathione synthesis and mitochondrial homeostasis^9,10^. ATM protein-deficient cells are hypersensitive to agents that induce DSBs and reactive oxygen species (ROS)^11^ and exhibit elevated intracellular ROS and oxidative protein, lipid, and DNA damage^12–15^. Additionally, ATM modulates cytokine and chemokine production in a cell type-specific fashion^16,17^ and is required for optimal inflammasome activation in response to microbial infections^18^. Thus, multiple defects in cellular and inflammatory responses to DNA damage, oxidative stress and infectious insults contribute to the pleiotropic phenotypes observed in A-T.

A-T patients with respiratory disease develop chronic progressive pulmonary complications with acute inflammatory alterations and radiographic changes^19^. Aberrant inflammatory responses in A-T are characterized by elevated percentages of neutrophils in the bronchoalveolar lavage (BAL) fluid^19^. Neutrophils from A-T patients produce elevated levels of the pro-inflammatory cytokine, IL-8, and have a prolonged lifespan compared to controls^17^. It is noteworthy that reduced lung function in A-T patients has been correlated with elevated serum levels of IL-8, as well as IL-6^20,21^. These findings suggest that defective pro-inflammatory responses intrinsic to ATM-deficient immune cells may exacerbate damage within lung tissues, which are highly sensitive to oxidative and DNA damage. However, the importance of ATM-deficient immune cells versus lung tissues in the initiation and progression of pulmonary complications in A-T has not yet been defined. Thus, in this study, we determine the impact of ATM-deficiency on the phenotypes of key inflammatory cells and define the specific contributions of ATM null immune cells to the inflammatory and pulmonary phenotypes in WT lungs.

There are currently no effective curative strategies for A-T, and thus far, effective therapeutic interventions for the most debilitating A-T phenotypes have remained elusive^22^. To identify potential treatments for the severe lung phenotypes in A-T, we targeted neutrophil-mediated inflammatory responses. To this end, we examined the impact of reparixin, a specific noncompetitive allosteric inhibitor of the C-X-C chemokine receptors, CXCR1 and CXCR2, on the pulmonary phenotypes in ATM-deficient mice^23^. The CXCL1, CXCL2 and IL-8/CXCL8 chemokines mediate neutrophil recruitment and activation via CXCR1/CXCR2^24–26^, and reparixin has been reported to inhibit neutrophil-mediated inflammatory responses in mouse models of acute lung injury and lung transplantation^23,27–29^. In patients undergoing cardiopulmonary bypass surgery, reparixin treatment significantly reduced neutrophils in the blood in comparison to placebo controls at the beginning, end and 1-h post-surgery^30^. Importantly, clinical trials have demonstrated that usage of reparixin in humans is safe and does not cause significant deleterious side effects^31,32^. In the current study, we find that reparixin attenuates key inflammatory phenotypes in our preclinical mouse model of A-T lung disease, thereby indicating that antagonism of CXCR1/CXCR2 may represent an effective therapeutic strategy for this frequent cause of death in A-T patients.

## Results

### Pulmonary function and inflammation in response to bleomycin-induced lung injury in a mouse model of A-T

**Figure 1:**
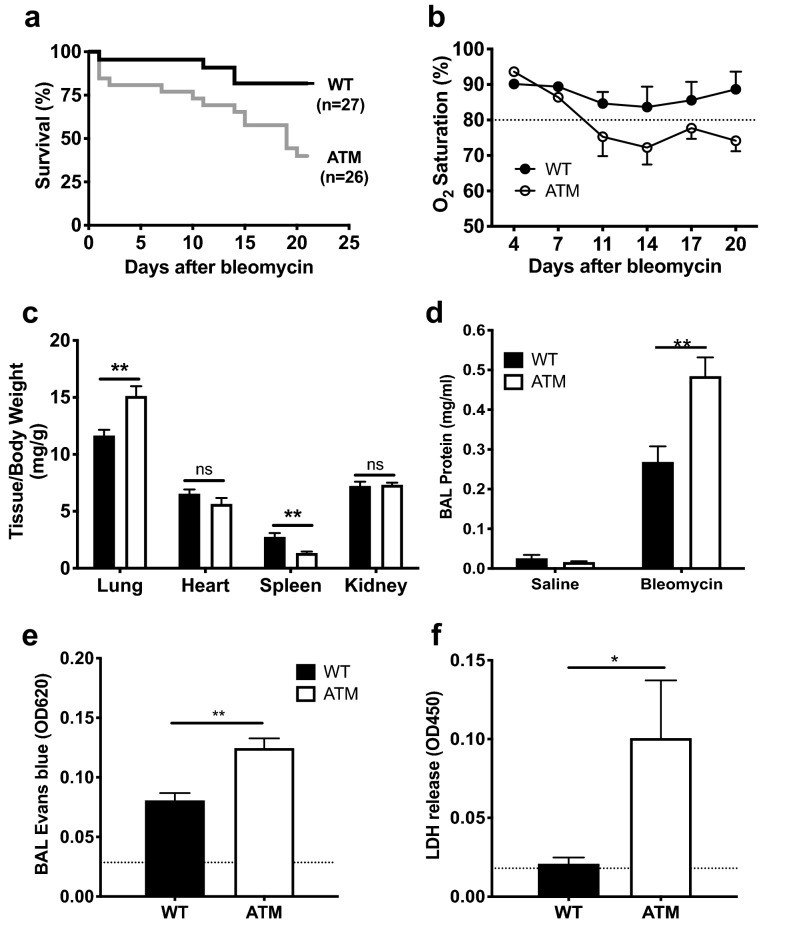
ATM-deficiency causes decreased survival and exacerbates lung injury upon bleomycin instillation. (**a**) Kaplan–Meier survival plot of ATM^∆/∆^ and WT mice instilled with 0.025U bleomycin. Survival was determined up to 21 days post-instillation (p = 0.006, log rank test). (**b**) Arterial oxygen saturation (%) was measured in conscious, unrestrained and non-sedated mice at the indicated days post-bleomycin administration. Dotted line, 80% O_2_ saturation, lower values indicate a state of severe hypoxia. (**c**) Ratios of wet weights of the indicated organs (mg) to total body weights (g) of bleomycin instilled mice were determined. (**d**) Protein concentrations (mg/mL) of the BALs were determined using the Bradford assay. (**e**) As a measure of vascular leakage, Evans blue dye (50 mg/kg) was injected via the tail vein and levels of dye in BAL fluid were quantitated after 3 h (A620). Dotted line, Evans blue levels in BAL fluid of saline-treated control animals. (**f**) Levels of released lactate dehydrogenase (LDH) in BAL fluid were assessed by the LDH-cytotoxicity assay (Roche). Dotted line, LDH in BAL fluid of saline-treated control animals. WT mice, black bars; ATM^∆/∆^ mice, white bars. Each experiment was performed on a minimum of 5 animals from at least 2 independent experiments at d21 post-bleomycin instillation. Bars, mean ± SEM. *P ≤ 0.05; **P ≤ 0.01.

We induced acute lung injury via oropharyngeal instillation of the oxidant, bleomycin, in ATM-deficient (ATM^∆/∆^) and wild-type (WT) mice. The sequence of pathological responses induced by bleomycin has been widely studied, and this experimental system represents one of the best-characterized preclinical models to study the progression of interstitial lung disease following an acute insult^33–35^. The ATM-deficient mouse model used in the current study harbors a gene-targeted deletion of exons 57–58, which encode the core kinase domain, and mimics disease alleles found in A-T patients^36–38^. Previous studies have reported that acute lung injury of ATM-deficient mice via administration of hydrocholoric acid or bleomycin results in elevated inflammatory responses^39,40^. Distinct A-T patient gene mutations and different knock-in and knock-out targeted mutations in the murine *ATM* gene cause a range of cellular and organismal phenotypes that vary in severity^36,41–45^. In this regard, the previously studied A-T mouse model harboring a different targeted *ATM* allele exhibited a significantly earlier onset of lymphoid tumors and shorter mean survival compared to our ATM-deficient mouse model^44,46^. Therefore, we determined the range and severity of lung phenotypes in tumor-free animals using our mouse model of A-T lung disease. We observed that bleomycin-instilled ATM^∆/∆^ mice exhibited markedly reduced survival, oxygen saturation and body weight compared to WT mice (Fig. 1a,b, Supplemental Fig. 1A). No differences in breath and heart rates between the ATM^∆/∆^ and WT cohorts were observed, suggesting that impaired respiratory or heart muscle strength did not significantly affect the observed phenotypes (Supplemental Fig. 1B,C).

Bleomycin-induced acute lung injury is characterized by tissue damage and increased permeability of the alveolar-capillary barrier resulting in lung edema with protein-rich fluid, which impairs arterial oxygenation. We evaluated the impact of ATM-deficiency on pulmonary fluid accumulation by determining the ratio of wet lung weight to body weight. ATM-deficient lungs from bleomycin-treated mice exhibited significantly higher wet weights compared to WT controls (Fig. 1c). We also observed a significant decrease in spleen weights, which is consistent with the known roles for ATM in antigen receptor gene rearrangements during lymphocyte development^47^. In comparison, we did not observe any differences in heart or kidney weights (Fig. 1c).

To further assess the impact of ATM-deficiency on lung edema, we measured protein levels in BAL fluid isolated from the experimental cohorts. We observed an approximately twofold increase in protein concentrations in bleomycin-instilled ATM^∆/∆^ BAL fluid compared to WT controls (Fig. 1d). We next examined the permeability of the vasculature by introducing Evans blue dye into bleomycin-treated animals via intravenous tail vein injection. Evans Blue has a high affinity for serum albumin and remains within blood vessels; however, in pathologic conditions that promote increased vascular permeability, extravasation of Evans Blue into tissues can be observed. We observed that ATM-deficiency increased levels of Evans Blue in the BAL fluid at 3 h post-injection compared to controls (Fig. 1e), whereas equivalent levels of dye were observed in plasma (Supplemental Fig. 1D). These findings indicate that not only the vasculature but also the lung epithelium were damaged in ATM^∆/∆^ lungs, thereby leading to hemorrhage and leakage of Evans Blue into the BAL fluid. We also found significantly higher lactate dehydrogenase, an indicator of cell injury and hemolysis^48^, in bleomycin-instilled ATM-deficient BALs (Fig. 1f). Additionally, we consistently observed between three- and tenfold higher numbers of red blood cells and increased hemoglobin concentrations in ATM-deficient BAL fluid compared to controls after bleomycin instillation (Table 1). These findings indicate that ATM-deficiency results in increased pulmonary edema, vascular leakage and hemorrhage within the lung tissues in response to acute lung injury.

**Table 1:** BAL RBC counts and hemoglobin levels in bleomycin-instilled animals.

Genotype	BAL RBC counts (× 10^6^)^a^	BAL hemoglobin (mg/mL)^a^	Neutrophils (% of BAL cells)^a^
WT (n = 5)	2.15 ± 0.32	0.04 ± 0.01	0.94 + 0.72 (n = 9)
ATM^∆/∆^ (n = 9)	7.45 ± 4.0	0.08 ± 0.02	25.54 + 6.02 (n = 7)
WT → WT (n = 4)	1.30 ± 0.07	0.08 ± 0.02	0.94 ± 0.49
ATM → WT (n = 7)	3.30 ± 0.32	0.27 ± 0.1	10.39 ± 5.16

^a^Mean ± SEM.

**Figure 2:**
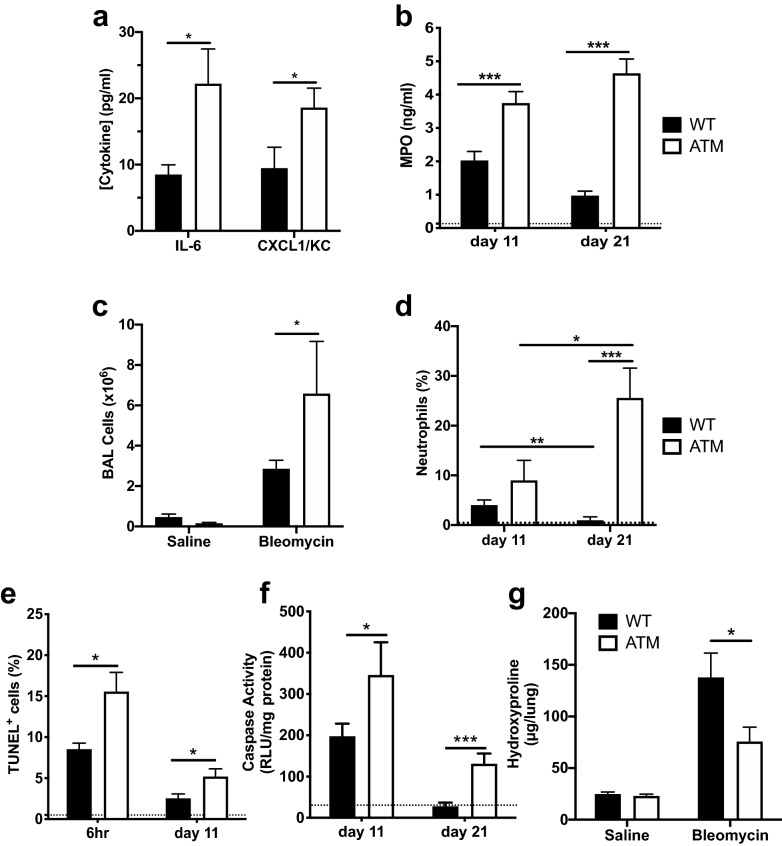
ATM-deficiency leads to exaggerated inflammatory responses and increased apoptosis in response to acute lung injury. (**a**) Levels (pg/mL) of the pro-inflammatory cytokine, IL-6, and the neutrophil attractant chemokine, KC/CXCL1, were quantitated in BAL supernatants from bleomycin treated WT and ATM-deficient mice at d21 by ELISA using mouse recombinant proteins as standards. (**b**) BAL myeloperoxidase levels (MPO, ng/mL) were assessed by ELISA at d11 and d21 post-bleomycin instillation. Dotted line, MPO levels in BAL fluid of saline-treated control animals. (**c**) Live BAL cells (× 10^6^) were quantitated by counting using a hemocytometer and staining with the viability dye, Trypan Blue. Saline vs. bleomycin cell numbers: P ≤ 0.001 for WT; P ≤ 0.05 for ATM^∆/∆^. (**d**) Percentages of neutrophils in the BALs from WT and ATM^∆/∆^ mice were assessed by flow cytometry at d11 and d21 post-bleomycin. dotted line, % neutrophils in control saline-instilled BALs. Number of animals for A-D: d11: WT, n = 11; ATM^∆/∆^, n = 8; d21: WT, n = 11; ATM^∆/∆^, n = 5. (**e**) Percentages of TUNEL positive cells in 10 arbitrarily selected microscopic fields of lung sections of WT and ATM deficient mice at 6 h and d11 post-bleomycin instillation were quantitated using ImageJ software in a blinded manner. Number of animals: 6 h: WT, n = 5; ATM^∆/∆^, n = 7; d11: WT, n = 10; ATM^∆/∆^, n = 10. Dotted line, % TUNEL + cells in control saline-instilled lungs. (**f**) Caspase 3/7 activity in lung homogenates from WT and ATM^∆/∆^ mice at d11 and d21 post-bleomycin instillation was determined using a luminescence-based assay (Promega). Number of animals: d11: WT, n = 12; ATM^∆/∆^, n = 7; d21: WT, n = 10; ATM^∆/∆^, n = 8. Bars, mean ± SEM; *P ≤ 0.05; ***P ≤ 0.001. Dotted line, caspase activity in control saline-instilled lungs. (**g**) Levels of hydroxyproline (μg/lung) were quantitated in lung homogenates using the acid hydrolysis Woessner method at d21 post-bleomycin. WT + saline, n = 6; WT + BLM, n = 11; ATM^∆/∆^ + saline, n = 5; ATM^∆/∆^ + BLM, n = 9; bars, mean ± SEM; *p ≤ 0.05.

We next probed the inflammatory responses in lung tissues and BAL fluid in bleomycin-instilled ATM^∆/∆^ and WT mice. Significantly higher levels of the murine functional homolog of IL-8, CXCL1/KC, and the pro-inflammatory cytokine, IL-6, were detected in ATM-deficient BALs, which persisted to d21 post-instillation (Fig. 2a). Myeloperoxidase (MPO) is the most abundant enzyme in neutrophils and functions to convert hydrogen peroxide into highly reactive, hypochlorous acid^49^. ATM-deficiency resulted in an approximately twofold increase in MPO levels in comparison to WT controls at d11 post bleomycin-instillation. At d21, MPO in WT BALs decreased, as expected for the subsidence of the inflammatory response. However, MPO in ATM-deficient BALs further increased and levels were approximately fivefold higher compared to controls at the later time post injury (Fig. 2b).

We observed increased recruitment of inflammatory cells in ATM-deficient and WT BALs in response to bleomycin-induced injury, and the overall number of cells was significantly higher in ATM-deficient BALs (Fig. 2c). Within these populations of BAL cells, the percentage of neutrophils in ATM-deficient lungs was consistently elevated at d11 compared to WT controls. Strikingly, at d21, neutrophil recruitment to ATM^∆/∆^ lungs had further increased (Fig. 2d); whereas, in sharp contrast, the neutrophil influx significantly decreased in WT BALs, to nearly the levels observed in unchallenged lungs (Fig. 2d). Thus, inflammatory responses are substantially elevated in ATM-deficient lungs, and the aberrant responses persist beyond the normal period of inflammation.

A dysregulated and exaggerated inflammatory response and neutrophilia can cause excessive tissue damage due to the production of deleterious reactive species. We examined the extent of damage within lung tissues using the TUNEL assay, which detects fragmented DNA, a characteristic feature of apoptotic cells. We observed a significantly higher percentage of TUNEL-positive cells in bleomycin-treated ATM^∆/∆^ lungs at 6 h and d11 post-instillation compared to controls (Fig. 2e). Enzymatic assays for cleaved caspase 3/7 activity revealed that lung injury resulted in significantly higher levels of caspase activity in ATM-deficient lung tissues (Fig. 2f). Again, it is notable that caspase activity levels remained elevated in lung homogenates from ATM-deficient mice up to d21 after bleomycin exposure. Together, these results indicate that lung parenchyma in ATM-deficient mice is highly susceptible to bleomycin-induced injury and apoptosis, and the damage persists for a prolonged time beyond the initial insult.

The inflammatory phase of bleomycin-induced pulmonary disease is followed by fibrosis, which is characterized by excessive scarring and accumulation of collagen in the lungs^34^. The fibrotic responses in bleomycin-injured ATM^∆/∆^ lungs were assessed by examining the extent of collagen deposition in Masson’s trichrome stained histologic sections of lung tissues. We observed that the fibrosis scores for ATM-deficient mice were consistently lower than those for WT mice at d21 post-bleomycin instillation, although the scores did not reach statistical significance (Supplemental Fig. 2). We also quantitated the levels of hydroxyproline, a major component of collagen, in homogenized lung tissues as an index of interstitial collagen deposition and fibrosis^50^. We found that ATM-deficient lungs contained significantly lower hydroxyproline levels compared to WT lungs at d21 post injury (Fig. 2g). The reduced collagen deposition in ATM null lungs at d21, a time point when excessive fibrosis is generally observed in wildtype animals, suggests that the prolonged inflammatory responses in ATM^∆/∆^ lungs impact disease progression and initiation of fibrotic responses.

### Transfer of ATM deficient bone marrow to WT recipients results in defects in lung injury and repair

**Figure 3:**
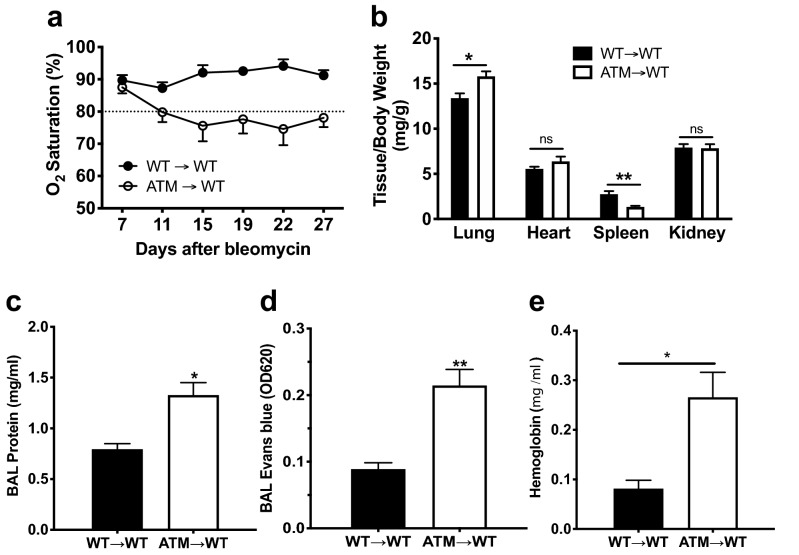
Transplantation of ATM-deficient bone marrow recapitulates acute lung injury phenotypes in chimeric mice. All experiments were performed on lethally irradiated WT recipients of WT (black bars, WT → WT) or ATM^∆/∆^ (white bars, ATM → WT) bone marrow. Chimeric mice were bleomycin instilled (0.025U) five weeks post BMT and followed for a period of 28 days. (**a**) Arterial oxygen saturation was measured on WT → WT and ATM → WT BMT chimeras. A minimum of 5 animals of each genotype was examined; O_2_ saturation percentages (mean ± SEM) are plotted as a function of time (days post-bleomycin). (**b**) Ratios of wet weights of the indicated organs (mg) to total body weights (g) of bleomycin instilled mice were determined. (**c**) Protein concentrations (mg/mL) of the BALs were determined using the Bradford assay. (**d**) As a measure of vascular leakage, Evans blue dye (50 mg/kg) was injected via the tail vein and levels of dye in BAL fluid after 3 h by measuring absorbance at 620 nm. (**e**) Hemoglobin concentrations (mg/mL) in cell-free BAL fluid supernatants were quantitated using Drabkin’s hemoglobin assay and spectrophotometric analysis at A540. Number of animals: WT → WT, n = 5; ATM → WT, n = 7. Bars, mean ± SEM; *P ≤ 0.05; **P ≤ 0.01.

To assess the specific contribution of hematopoietic cells to the severe phenotypes observed in the bleomycin-instilled lungs of ATM-deficient mice, we adoptively transferred ATM-deficient (ATM > WT) or WT (WT > WT) BM cells from 3 to 5 week old mice into lethally irradiated WT mice. At 5 weeks post-transplantation, bleomycin was administered to the reconstituted mice, and the phenotypes of the recipient mice were assessed at d28 post-instillation. The numbers of the reconstituted hematopoietic cell populations were comparable to those observed in the BALs of WT and ATM^∆/∆^ germline deleted animals (Supplemental Fig. 3A). We found that, similar to the ATM^∆/∆^ germline mice, bleomycin-induced lung injury resulted in lower arterial oxygen saturation in WT recipients of ATM^∆/∆^ bone marrow (Fig. 3a). The lung weights of the ATM > WT chimeras were higher in comparison to WT > WT chimeras, indicating increased edema associated with transfer of the ATM^∆/∆^ immune cells (Fig. 3b). Indeed, significant increases in total BAL protein, Evans blue, hemoglobin and red blood cells were observed in recipients of ATM^∆/∆^ bone marrow compared to those receiving WT bone marrow (Fig. 3c–e, Table 1). We also consistently observed higher neutrophil recruitment in the BALs of bleomycin treated ATM > WT chimeras (WT > WT, 0.94% ± 0.49; ATM > WT, 10.39% ± 5.16; Table 1). Despite the phenotypes demonstrating decreased lung function and increased vascular leakage, similar survival rates were observed between the WT > WT and ATM > WT mice, thereby indicating that lung intrinsic functions of ATM also contribute to the pathophysiology of the pulmonary phenotypes (Supplemental Fig. 3b). Nonetheless, these findings demonstrate that ATM^∆/∆^ immune cells play a major role in exacerbating acute lung injury that results in vascular leakage, edema and decreased pulmonary function.

**Figure 4:**
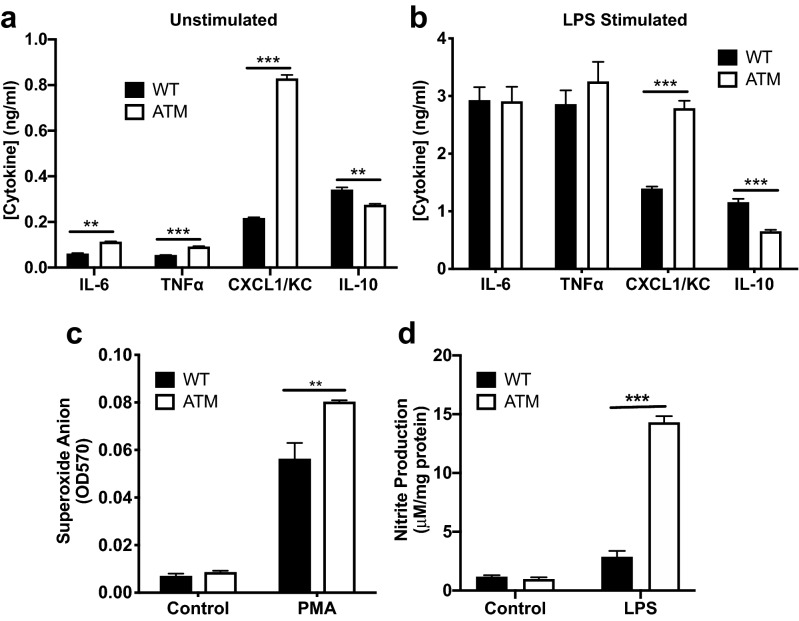
ATM-deficient macrophages exhibit aberrant production of chemokines and cytokines and elevated levels of reactive species. (**a**) Primary macrophages derived from bone marrow isolated from WT or ATM^∆/∆^ mice were cultured, and levels of the indicated cytokines and chemokines were measured in cell culture supernatants by ELISA. (**b**) Bone marrow derived macrophages were stimulated with LPS (1 μg/mL), and the cytokine and chemokine levels were determined at 24 hr post-stimulation. (**c**) Intracellular superoxide anion (O_2_^−^) in WT and ATM^∆/∆^ primary macrophages was quantitated in PMA (1 μg/mL) + ionomycin (1 μg/mL) stimulated cultures (30 min) and unstimulated (control) cultures using a modified nitroblue tetrazolium assay. (**d**) NO_2_ levels, as a measure of RNS, were quantitated in supernatants from cultured unstimulated and LPS-stimulated WT or ATM^∆/∆^ primary macrophages using the Greiss assay. P ≤ 0.01 for unstimulated vs. stimulated cultures in (**c**,**d**). Data were obtained from a minimum of 3 independent experiments; Bars, mean ± SEM; **P ≤ 0.01; ***P ≤ 0.001.

Macrophages have critical functions in maintaining lung homeostasis, including mediating the initiation and resolution of inflammatory responses through production of cytokines, chemokines, toxic mediators and reactive species^51^. Both bone marrow derived monocytes and alveolar residing macrophages contribute to the inflammatory response^52^. To assess the impact of ATM-deficiency on critical functions of primary macrophages, we examined the phenotypes of bone marrow-derived primary macrophages (BMDM). We observed that unstimulated ATM-deficient macrophages produced significantly elevated levels of the pro-inflammatory molecules, IL-6, TNF-α and CXCL1/KC, and decreased levels of the anti-inflammatory cytokine, IL-10, compared to WT controls (Fig. 4a). LPS-stimulation markedly increased production of the chemokines and cytokines in both WT and ATM^∆/∆^ BMDMs (Fig. 4b). Stimulated ATM-deficient and WT macrophages produced similar levels of IL-6 and TNF-α. In contrast, significantly elevated levels of the neutrophil chemoattractant, CXCL1/KC, and lower amounts of IL-10 were produced by ATM^∆/∆^ macrophages compared to controls (Fig. 4b). Activated macrophages generate ROS and reactive nitrogen species (RNS), which, if unregulated, can cause excessive tissue damage. Upon mitogen stimulation, ATM-deficient macrophages produced markedly higher levels of intracellular ROS and RNS (Fig. 4c,d). Together, these results demonstrate that ATM-deficient immune cells exhibit aberrant inflammatory responses and likely contribute to the pathophysiology of A-T lung disease.

### The CXCR1/CXCR2 antagonist, reparixin, attenuates pulmonary phenotypes in ATM null mice

Neutrophils play central roles in driving pulmonary inflammation and elevated numbers of neutrophils are associated with increased severity of disease and mortality^53–56^. Therapeutic strategies that inhibit neutrophil recruitment have been demonstrated to attenuate inflammatory responses and improve pulmonary disease phenotypes in preclinical mouse models and clinical trials^57–61^. Thus, based on our observations of neutrophilia and increased production of the neutrophil chemoattractant, CXCL1/KC, in ATM-deficient lungs, we hypothesized that inhibition of neutrophil recruitment could attenuate pulmonary inflammation and substantially improve the observed lung pathologies. CXCR2 is the primary chemokine receptor for CXCL1/KC, and the CXCR2 and CXCR1 chemokine receptors play primary roles in neutrophil recruitment and activation in response to lung injury^61,62^. Thus, we treated ATM-deficient mice with reparixin, a specific noncompetitive allosteric inhibitor of CXCR1/CXCR2 shown to inhibit neutrophil-driven inflammatory responses in mouse models and patients^23,27–32^.

**Figure 5:**
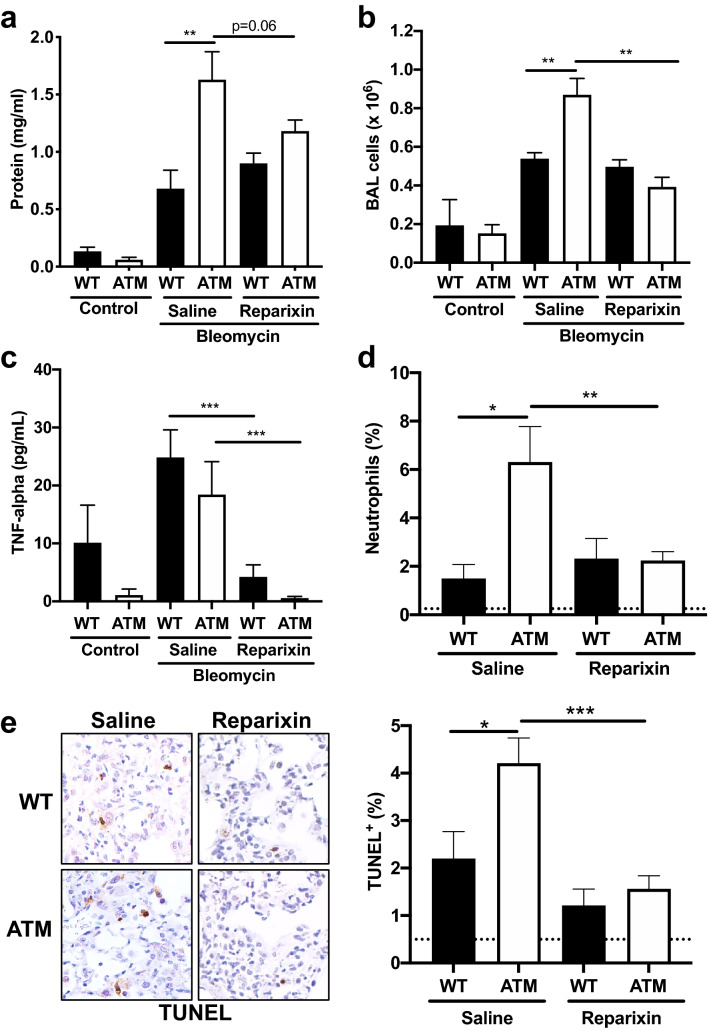
Impact of reparixin treatment on aberrant inflammatory responses in ATM-deficient mice. Bleomycin-instilled ATM^∆/∆^ and WT mice received intraperitoneal injections of either reparixin or saline twice daily for a period of 7 days. BAL fluid collection and analysis occurred on d14 post-instillation. (**a**) Protein concentrations in BAL fluid were determined using the bicinchoninic acid assay at d14 post-instillation. (**b**) Live BAL cells (× 10^6^) were quantitated by counting using a hemocytometer and staining with the viability dye, Trypan Blue. (**c**) TNF-α levels were measured in BALs via ELISA from reparixin treated WT and ATM^∆/∆^ mice and controls. (**d**) Percentages of neutrophils in the BALs from reparixin or saline treated WT and ATM^∆/∆^ mice were assessed by flow cytometry at d14 post-bleomycin. Dotted line, % neutrophils in control saline-instilled lungs. (**e**) TUNEL positive cells in 6 arbitrarily selected microscopic fields of lung sections of WT and ATM^∆/∆^ mice at d14 post-bleomycin were quantitated. Number of animals: Saline: WT, n = 5; ATM^∆/∆^, n = 7; Reparixin: WT, n = 10; ATM^∆/∆^, n = 10. Dotted line, % TUNEL + cells in control saline-instilled lungs. Bars, mean ± SEM; *P ≤ 0.05; **P ≤ 0.01; ***P ≤ 0.001.

WT and ATM^∆/∆^ mice exposed to oropharyngeal bleomycin were treated with either reparixin or normal saline and analyzed 14 days following bleomycin exposure. Administration of the CXCR1/CXCR2 antagonist had a significant impact on several relevant pulmonary phenotypes in the ATM-deficient lungs. We observed a significant reduction in the number of inflammatory cells and a trend towards reduced protein levels in the BAL fluid of bleomycin treated ATM-deficient mice following administration of reparixin compared to controls (Fig. 5a,b). CXCL1/KC induces production of TNF-α, which in turn mediates neutrophil influx^63^. Following bleomycin exposure, antagonizing CXCR1/CXCR2 with reparixin resulted in a statistically significant reduction of the pro-inflammatory cytokine, TNF-α, in the BALs of both WT and ATM^∆/∆^ mice (Fig. 5c). We also observed a concomitant decrease in neutrophil influx in the BALs of bleomycin-instilled ATM-deficient mice, with the percentage of neutrophils reduced to levels similar to those found in WT BALs (Fig. 5d). We next examined the impact of reparixin administration on pulmonary cellular apoptosis. The percentage of TUNEL positive cells in bleomycin instilled ATM^∆/∆^ lung tissues was significantly reduced in reparixin treated animals to a level comparable to that observed in WT lungs (Fig. 5e). Together, these findings suggest that CXCR1/CXCR2 blockage has therapeutic benefit in attenuating key pulmonary phenotypes caused by ATM-deficiency, including production of the pro-inflammatory cytokine, TNF-α, neutrophilia and lung injury.

## Discussion

Lung disease is a major cause of morbidity and mortality in A-T patients^5^; however, the underlying mechanisms and critical cell types involved in the development of pulmonary complications have not been fully defined. In this study, we examined the phenotypes induced by acute lung injury in ATM-deficient mice and probed the contributions of ATM^∆/∆^ immune cells in the etiology of A-T lung disease. Additionally, because there are currently no effective treatments for this fatal disease phenotype, we sought to identify potential targets for therapeutic intervention and take advantage of these findings to test the efficacy of a targeted therapy. Herein, we demonstrate that bleomycin-induced lung injury induces elevated inflammatory responses in ATM-deficient mice compared to controls, including higher BAL levels of MPO and pro-inflammatory chemokines/cytokines and elevated neutrophil influx. Notably, these phenotypes persisted until day 21 post bleomycin instillation, a markedly longer time compared to WT mice in which inflammatory responses generally subside by approximately day 14^34,64^. We also observed persistent and elevated apoptosis associated with vascular leakage, epithelial damage, hemorrhage and edema in ATM^∆/∆^ lungs. Prolonged or nonresolving inflammation can cause tissue damage that potentiates further inflammation and can lead to pathologies, including chronic pulmonary diseases^65,66^. Thus, the exaggerated and sustained inflammatory responses and increased tissue damage caused by ATM-deficiency likely underlie the pulmonary complications observed in A-T patients.

In our study, we established that ATM-deficient immune cells play critical roles in the pathophysiology of the lung phenotypes. Transfer of ATM^∆/∆^ bone marrow into WT recipient mice caused aberrant inflammatory responses, similar to those observed in germline deleted ATM-null mice following bleomycin instillation, including elevated neutrophil influx, vascular leakage, epithelial damage and reduced oxygen saturation. We also demonstrated that activated ATM-deficient macrophages exhibit phenotypes that could promote aberrant inflammation and tissue damage. In this regard, unstimulated ATM^∆/∆^ macrophages produce increased levels of the pro-inflammatory chemokines, IL-6, TNF-α and CXCL1/KC, and upon stimulation, significantly higher levels of CXCL1/KC are produced in comparison to WT macrophages. Moreover, lower levels of the anti-inflammatory cytokine, IL-10, are produced in both unstimulated and stimulated ATM^∆/∆^ macrophages, thereby generating a pro-inflammatory environment via an imbalance of cytokine/chemokine production. Stimulated ATM^∆/∆^ macrophages were also found to produce increased levels of ROS and RNS. As ATM-deficient cells are inherently hypersensitive to oxidative damage^67^, elevated levels of reactive species and ROS/RNS-induced DNA damage would be predicted to contribute to the high levels of apoptosis observed in ATM^∆/∆^ lungs. Thus, our findings indicate that the lung intrinsic functions of ATM prevent excessive tissue damage, which is potentially exacerbated by prolonged exposure to damaging reactive species. The importance of the protective functions of ATM within lung tissues is further supported by our observations that despite the exaggerated inflammatory responses observed in the ATM > WT chimeric mice, these mice did not exhibit increased mortality, and the phenotypes were less severe compared to the ATM-null mice.

We propose that the constellation of immune system and lung intrinsic phenotypes caused by ATM-deficiency promotes a detrimental cycle involving (1) enhanced tissue damage and cell death in response to an insult, (2) induction of exaggerated, prolonged inflammatory responses, (3) additional tissue damage, and (4) impairment or delay of proper resolution and repair. In human patients, chronic inflammatory pulmonary diseases, such as chronic obstructive pulmonary disease, lung fibrosis and bronchiectasis, are characterized by neutrophil dysregulation that causes damaging inflammation^53,56^. Thus, our findings suggest that the ATM-deficiency perpetuates the impact of an initial lung injury by promoting a cycle of chronic inflammation and tissue damage that ultimately result in lung disease in A-T patients, including bronchiectasis and consolidation^68^.

Inhibition of neutrophil recruitment has been demonstrated to decrease inflammation and improve lung function in preclinical models and clinical trials^57–62^; therefore, we evaluated the therapeutic impact of blocking CXCL1/KC-mediated neutrophil recruitment with the CXCR1/CXCR2 antagonist, reparixin. We demonstrated that reparixin treatment reduced neutrophil influx to ATM^∆/∆^ lungs and effectively prevented the extensive cell death observed in untreated animals. Although neutrophils represent a small proportion of cells within the alveolar space, indicating the possibility that additional immunologic cells may play an important role, such a reduction in a key component of the inflammatory response is nonetheless a promising finding. Intervention with reparixin also resulted in a marked reduction in production of the pro-inflammatory cytokine, TNF-α, which is produced upon binding of CXCL1/KC to the CXCR2 receptor. It is notable that TNF-α has been implicated in the development and propagation of severe respiratory disorders^69–71^, and therapeutic strategies involving TNF inhibition have had positive clinical outcomes in treatment of severe pulmonary conditions, including idiopathic pneumonia syndrome following bone marrow transplantation^72^. Thus, antagonizing CXCR1/CXCR2 with reparixin has multiple beneficial effects on the lung pathologies caused by ATM-deficiency via decreasing TNF-α production and reducing neutrophil recruitment and activation.

Overall, our studies support the notion that a CXCR1/CXCR2 blockade agent, such as reparixin, represents a promising therapeutic strategy for lung complications in A-T. The positive effects of reparixin on inhibiting neutrophil-driven inflammation and decreasing production of pro-inflammatory cytokines/chemokines significantly reduced tissue damage in ATM-deficient lungs. It is possible that other phenotypes caused by ATM-deficiency may be exacerbated by inflammation-induced oxidative stress and exhibit improvement upon reparixin treatment. Thus, it would be of interest to examine the long-term beneficial effects of reparixin on additional phenotypes in ATM-deficient animals, such as tumorigenesis and longevity. Clinical trials examining the efficacy of reparixin on preventing deleterious inflammatory conditions have reported positive outcomes without significant negative side effects^30–32,73^. To our knowledge, our studies provide the first potential therapeutic strategy for A-T lung disease. The findings establish a foundation for additional investigations aimed at further improving the phenotypic outcomes of this targeted treatment. To this end, studies using next generation small molecule inhibitors of CXCR1/CXCR2 and other targeted strategies, such as TNF-α therapeutics or novel inhibitors of neutrophil influx or activation, represent critical next steps toward preventing A-T lung disease.

## Materials and methods

### Mice

ATM^∆/∆^ mice were generated from Cre recombinase-dependent deletion of an ATM conditional targeted allele, which harbored loxP sites flanking exons 57 and 58 of the murine ATM locus^38^. ATM^∆/∆^ and wild-type (WT) littermates were obtained for analyses from interbreeding heterozygous ATM^+/∆^ mice (C57BL/6 × 129/SvJ). The genotypes of WT and ATM^∆/∆^ mice were confirmed by polymerase chain reaction (PCR) analysis performed on DNA prepared from the tails of 3-week-old animals. The mice were housed in a specific pathogen-free facility for immunocompromised animals. The animal protocols and procedures involving the experimental mice were reviewed and approved of by the University of Michigan Institutional Animal Use & Care Committee. The protocol is annually reviewed to ensure that the project is in accordance with local, state and federal laws or regulations for humane treatment of animals, the USDA Animal Welfare Act/Regulations, and the Public Health Service Policy.

### Bleomycin-induced lung injury and pulmonary fibrosis as a model of A-T lung disease

Specific pathogen-free, 8- to 10-week-old homozygous ATM^∆/∆^ and WT mice with no obvious signs of thymic lymphoma received a single dose of 0.025 U of bleomycin sulfate (Bristol-Meyers Pharmaceuticals) dissolved in 50 μl of sterile saline via oropharyngeal instillation. Control mice received saline alone.

### PCR genotyping

Genotypes of the mice bearing the ATM^∆/∆^ or WT alleles were determined by PCR analysis with the following primers: P1: 5′-GCC CAT CCC GTC CAC AAT ATC TCT GC-3′, P2: 5′-CAT CCT TTA ATG TGC CTC CCT TCG CC-3′ and P3: 5′-ATG GCT TCG AGG TTG AGC GTA CTT-3′. Amplification of PCR products for ATM was performed by denaturation at 94 °C for 3 min and then 35 cycles of amplification at 94 °C for 45 s, 55 °C for 60 s, and 72 °C for 45 s, followed by 10 min extension at 72 °C.

### Bone marrow (BM) transplantation

BM chimeras were prepared as previously described with minor modification^74^. BM cells were collected from femurs and tibias of 3–5-week-old donor ATM-deficient or WT mice by aspiration and flushing. Ablation of recipient-derived hematopoietic stem cells (HSCs) was achieved by the administration of a fractionated 13-Gy dose of total body irradiation. Recipient mice were exposed to two doses of 6.5 Gy given 3 h apart using a ^137^Cs irradiator or an x-ray orthovoltage source, and then maintained on acidified water and autoclaved feed ad libitum. After irradiation, 5 × 10^6^ BM cells from ATM^∆/∆^ or WT mice in a volume of 200 μl sterile PBS were injected into the tail vein of recipient animals. Complete immune reconstitution is achieved 5 weeks following the infusion of BM cells into total body irradiation recipients^74^.

### Analysis of adoptive transfer of bone marrow to irradiated recipients

The successful engraftment and reconstitution of ATM^∆/∆^ and WT bone marrow in irradiated WT recipients was determined at 28 days after bone marrow transplantation. The spleens were dissected and dissociated into single cell suspensions, and peripheral blood was collected from the retro-orbital vein. The bone marrow cells were flushed from the femurs of the animals with PBS. The red blood cells in the samples were lysed with a Tris-ammonium chloride buffer (Invitrogen). The percentage of ATM^∆/∆^ donor-derived cells in the bone marrow, spleen, and lung was assessed by semi-quantitative genomic PCR and FACS analysis of the reconstituted populations of cells.

### Administration of reparixin to bleomycin instilled animals

Pathogen-free female and male 8- to 10-week-old homozygous ATM^∆/∆^ and WT mice received a single dose of 0.025 U of bleomycin sulfate (Bristol-Meyers Pharmaceuticals) dissolved in 50 μl of sterile saline via oropharyngeal instillation on day 0. Control mice received sterile saline alone. Beginning 8 h post-bleomycin exposure experimental mice received intraperitoneal injections of either reparixin (Cayman Chemicals, dosed at 20 mg/kg, administered via a concentration of 2.5 mg/mL) or sterile saline (control mice) twice daily for a period of 7 days. Pulse oximetry readings and weights were obtained on days 7, 11, and 14 and the mice were euthanized and analyzed on d14 post bleomycin instillation.

### Pulse oximetry

To assess peripheral blood oxygen content in vivo, mice were monitored for the percentage of hemoglobin saturated with oxygen (pulse ox). Animals were either conscious or anaesthetized for pulse ox readings. The fur on the necks of the animals was removed by shaving, and the sensor collar was directly placed on the skin. Three consecutive sustained readings for at least 15 s were averaged using the MouseOx Small Animal Oxymeter (Starr Life Sciences, Oakmont, PA) connected to a computer equipped with MouseOx software (Starr Life Sciences; www.starrlifesciences.com).

### Collection of bronchoalveolar lavage fluid

Bronchoalveolar lavage (BAL) was performed with ten 1 mL-aliquots of PBS through a tracheal cannula following euthanasia. The lungs were then either homogenized for analysis or inflation fixed in 10% formalin for histologic examination.

### BAL cellular analysis

The total cell counts of freshly collected BAL fluid were determined using a hemocytometer before and after hypotonic lysis of red blood cells. The remaining BAL fluid was centrifuged at 1000×*g* to pellet cells, and the supernatants were stored at − 80 °C for further analyses. Cytospins of BAL samples were fixed and stained with the Quick-Diff staining kit (Harleco Co., Gibbstown, NJ) for leukocyte differential cell counts. BAL erythrocyte concentrations were calculated by subtracting the total leukocyte concentration from the total cell concentration for each sample. BAL cell concentrations were expressed as cells per mL of BAL fluid. Protein concentrations of the BAL fluid were determined by bicinchoninic acid assay (Pierce, Rockford, IL). Albumin concentrations in the BAL fluid were quantified by ELISA (Bethyl Laboratories, Montgomery, TX).

### Chemokine and myeloperoxidase measurement

CXCL1/KC, MPO, IL-6, and TNF-α concentrations were determined in BAL fluid using a standardized ELISA technique (R&D Systems, Minneapolis, MN). Recombinant murine proteins were used to generate standard curves.

### Flow cytometry

BAL cells were incubated with monoclonal antibodies to mouse CD4 (RM4-5), CD8α (53–6.7), B220 (CD45R), CD3ε (145-2C11), TCRβ (H57-597), TCRγδ (UC7-13D5), CD49b (DX5), NK1.1 (PK136), CD11b (M1/70), F4/80 (BM8), Gr1 (Ly6G; RB6-8C5), CD45 (30-F11), CD19 (1D3), IFN-γ (XMG 1.2), IL-4 (11B11), IL-10 (JES5-16E3), and IL-17 (TC11-18H10) that were conjugated to fluorescein, phycoerythrin, or allophycocyanin (BioLegend). Data were analyzed with the AccuriC6 cytometer (BD) and FlowJo software (TreeStar, www.flowjo.com).

### Evans blue permeability assays

The extent of vascular permeability was determined by measuring the ratio of Evans blue dye in the lung tissue versus the plasma, as previously described^75^. Animals were administered 200 μl of 20 mg/mL Evans blue (Sigma) dissolved in saline via tail vein injection. Three hours after injection, animals were exsanguinated and blood plasma collected. BAL was performed with 5 mL of PBS; and then the lungs were removed, weighed, and homogenized in 3 mL of PBS. To this lung homogenate, 2 volumes formamide was added to extract Evans blue and samples were incubated at 55–60 °C overnight. The supernatant was then clarified by centrifugation at 5000×*g* for 30 min. The Evans blue concentrations of the supernatants of lung homogenates were determined by a dual wavelength spectrophotometric method at 620 and 740 nm in order to correct for contaminating heme pigments, using the following formula: E620 (Evans blue) = E620 − (1.426 × E740 + 0.030). Evans blue levels in the BAL samples were measured at 620 nm.

### Biochemical assays

BAL fluid was analyzed for lactate dehydrogenase (LDH) content (Cytotoxicity Detection Kit; Roche Diagnostics), hemoglobin (Drabkin’s reagent, 30% Brij 35; Sigma), and iron (Assay Kit MAK025; Sigma). Lung tissue homogenates were analyzed per manufacturer’s instructions for caspase-3/7 activity (Caspase-Glo Assay; Promega).

### Masson’s trichrome staining

Formalin-fixed and paraffin-embedded lung sections (5 mm) were stained with Masson’s trichrome for the evaluation of collagen content and distribution. Fibrotic lung injury and pulmonary morphological changes was assessed by the semiquantitative morphological index (SMI) and scored between 1 and 4 (pathologic fibrosis score). SMI grades were uniformly grade 1 for saline-treated mice, that is no abnormal alveolar architecture, no thickening of alveolar septa, and intermediate collagen deposition. All analyses were performed on coded slides by two blinded observers. Ten fields of stained lung sections from 4 to 6 mice of each genotype were scored.

### Hydroxyproline quantification

Total lung collagen was measured by assaying lung hydroxyproline content of the right lung after hydrolysis with 6 N HCl, as previously described^76^. Hydroxyproline levels (μg/lung) were measured in triplicate on lungs from at least 5 mice of each genotype.

### TUNEL assay

Apoptosis in paraffin-embedded lung tissue samples was quantified by terminal deoxynucleotidyl transferase-mediated deoxyuridine triphosphate nick-end labeling (TUNEL)^77,78^. The TUNEL assay was performed on tissue sections as per the manufacturer’s protocol (ApoTag Peroxidase In Situ Apoptosis Detection Kit; Millipore), and the apoptotic cells were detected with 3,3′-diaminobenzidine tetrahydrochloride (DAB; Invitrogen) with hematoxylin counterstaining. For each section, at least six 20 × fields were chosen randomly. The numbers of TUNEL-positive and TUNEL-negative cells were quantified using ImageJ software (NIH) in a blinded manner. The percentage of TUNEL + cells in each section was calculated, and the mean ± SEM for each sample was determined. Tissue sections from a minimum of 5 animals from at least 2 independent experiments were analyzed.

### Derivation of primary bone marrow-derived macrophages (BMDM) for in vitro analyses

Bone marrow-derived primary macrophages were prepared as previously described^79,80^. Briefly, the bone marrow from the femurs of WT or ATM^∆/∆^ mice was dissociated, and the cells were cultured in DMEM media containing the supernatants from L929 cells (30% v/v) and 10% FBS. After 6 days, the adherent cells were harvested, counted and re-plated. The expanded macrophage populations were examined by flow cytometry, and naïve macrophages were identified as MHC class II-low, CD11c-low, CD11b-hi and F4/80-hi.

### Determination of reactive oxygen and nitrogen species

Naïve macrophages were seeded on 24-well tissue-culture treated plates at 1 × 10^5^ cells per well in 1 mL of DMEM containing 10% FBS and allowed to adhere overnight. LPS (1 μg/mL) were added, as indicated, and cells were incubated at 37 °C in 5% CO_2_. At 48 h post-stimulation, supernatants were collected and assessed for nitric oxide (NO) production using the Griess assay to determine nitrite (NO_2_^-^) levels, as described previously^81^. NO_2_ levels were determined by mixing 100 μl assay buffer (equal volumes of 1% sulfanilamide in 30% acetic acid and 0.1% N-(1-napthyl) ethylenediamine dihydrochloride in 60% acetic acid) with 50 μl of cell supernatants in a 96-well plate. The absorbances at 570 nm of the samples were compared to NaNO_2_ standards, which were included in every assay. Intracellular superoxide anion (O_2_^-^) levels were measured in cultures stimulated with PMA (1 μg/mL) + ionomycin (1 μg/mL) using the modified nitroblue tetrazolium (NBT) assay, as previously described^82^. In brief, NBT (0.33 mg/mL) in media containing PMA + ionomycin was added to cells for 30 min. The cells were washed 2 times with PBS then fixed in methanol. The cells were permeabilized with 2 M KOH, and the blue formazan was solubilized in DMSO, then the absorbance at 570 nm was determined.

### Statistical analysis

Statistical differences between groups were determined using the ANOVA or two-tailed t tests using GraphPad Prism (version 8.0.0, GraphPad Software, San Diego, California USA, www.graphpad.com). Differences were considered significant at P ≤ 0.05. All experiments were conducted 3 or more independent times or with samples collected from at least 3 different mice from 2 or more independent experiments.

### Ethics statement

All procedures involving mice were approved by the University of Michigan Committee on the Use and Care of Animals.

## Supplementary information

Supplementary information.

## Abbreviations

A-T: Ataxia-telangiectasia
ATM: Ataxia-telangiectasia mutated
WT: Wild-type
KO: Knockout
DDR: DNA damage response
BAL: Bronchoalveolar lavage
MPO: Myeloperoxidase
KC: Keratinocyte-derived chemokine, also called CXCL1
CXCL1: C-X-C motif chemokine ligand 1
CXCL2: C-X-C motif chemokine ligand 2
TNF: Tumor necrosis factor
IL-6: Interleukin-6
TUNEL: Terminal deoxynucleotidyl transferase dUTP nick end labeling
LDH: Lactate dehydrogenase
ALI: Acute lung injury
PMN: Polymorphonuclear leukocytes
RBC: Red blood cell
BM: Bone marrow
BMT: Bone marrow transplantation
ILD: Interstitial lung disease
HSC: Hematopoietic stem cells
