# Supplementary material for: Elevated inflammatory responses and targeted therapeutic intervention in a preclinical mouse model of ataxia-telangiectasia lung disease
Source: Sci Rep. 2021 Feb 19;11:4268. doi: 10.1038/s41598-021-83531-3 (PMC7895952; doi:10.1038/s41598-021-83531-3)

Elevated inflammatory responses and targeted therapeutic intervention in a preclinical mouse model of ataxia-telangiectasia lung disease

Rudel A. Saunders[1,#], Thomas F. Michniacki[2,#], Courtney Hames[4], Hilary A. Moale[1], Carol Wilke[3], Molly E. Kuo[4], Johnathan Nguyen[4], Andrea J. Hartlerode[5], Bethany B. Moore[1,3], and JoAnn M. Sekiguchi*[1,4]

Departments of Internal Medicine[1], Pediatric Hematology/Oncology[2], Microbiology and Immunology[3], Human Genetics[4], Pathology[5], University of Michigan, Ann Arbor, Michigan

#Equal contribution

*Corresponding author:
Dr. JoAnn M. Sekiguchi
University of Michigan
109 Zina Pitcher Place
2063 BSRB, Box 2200
Ann Arbor, MI 48109
Email: sekiguch@med.umich.edu
(734) 764-9514 (office)
(734) 763-2162 (FAX)

Supplementary Figure Legends

Supplementary Figure 1. Body weight, breath and heart rates and pulmonary vascular leackage in bleomycin-instilled ATM-deficient animals. A. WT and ATM$^{\Delta/\Delta}$ mice were weighed at d0 and d21 post-bleomycin administration, and the percent weight change was determined. **B and C.** Breath rates (breaths per minute, brpm; **B**) and heart rates (beats per minute, bpm, **C**) of bleomycin-instilled WT and ATM$^{\Delta/\Delta}$ mice were measured at the indicated days post-instillation using the MouseOx Small Animal Oxymeter system. **D.** Levels of Evans blue dye injected into the tail vein in blood plasma were determined at 3 hr post i.v. injection. OD620 was determined after correction for heme pigments. A minimum of 5 animals of each genotype was analyzed for every assay; mean $\pm$ SEM. No significant differences were observed.

Supplementary Figure 2. Lower collagen deposition in the lungs of ATM-deficient mice. A. Masson's trichrome staining of left lung tissue sections from WT and ATM-deficient mice treated with bleomycin or saline, as indicated. Magnification 1000X. **B.** Fibrosis scores for trichrome stained tissue sections from bleomycin- and saline-treated WT and ATM$^{\Delta/\Delta}$ lungs at d21. A minimum of 10 histologic fields was scored (1-4, 1= no apparent changes in morphology in any field) in a blinded manner by at least 2 independent investigators; 4-6 mice of each genotype were analyzed. Mean scores $\pm$ SEM.

Supplementary Figure 3. Analysis of BAL cell populations in recipients of WT and ATM$^{\Delta/\Delta}$ bone marrow. A. Cell populations of BALs isolated from lethally irradiated WT recipients of WT and ATM$^{\Delta/\Delta}$ bone marrow were analyzed at 5 weeks post transplantation by flow cytometry to determine the extent of reconstitution. No significant differences in the

percentages of the indicated reconstituted cell types were observed. Mean ± SEM, Number of animals: WT→WT, n=5; ATM→WT, n=7. **B.** Survival curves of lethally irradiated WT recipients of WT (WT→WT) or $ATM^{\Delta/\Delta}$ (ATM→WT) bone marrow. Chimeric mice were bleomycin instilled (0.025U) five weeks post BMT and followed for a period of 28d.

Supplementary Figure 1

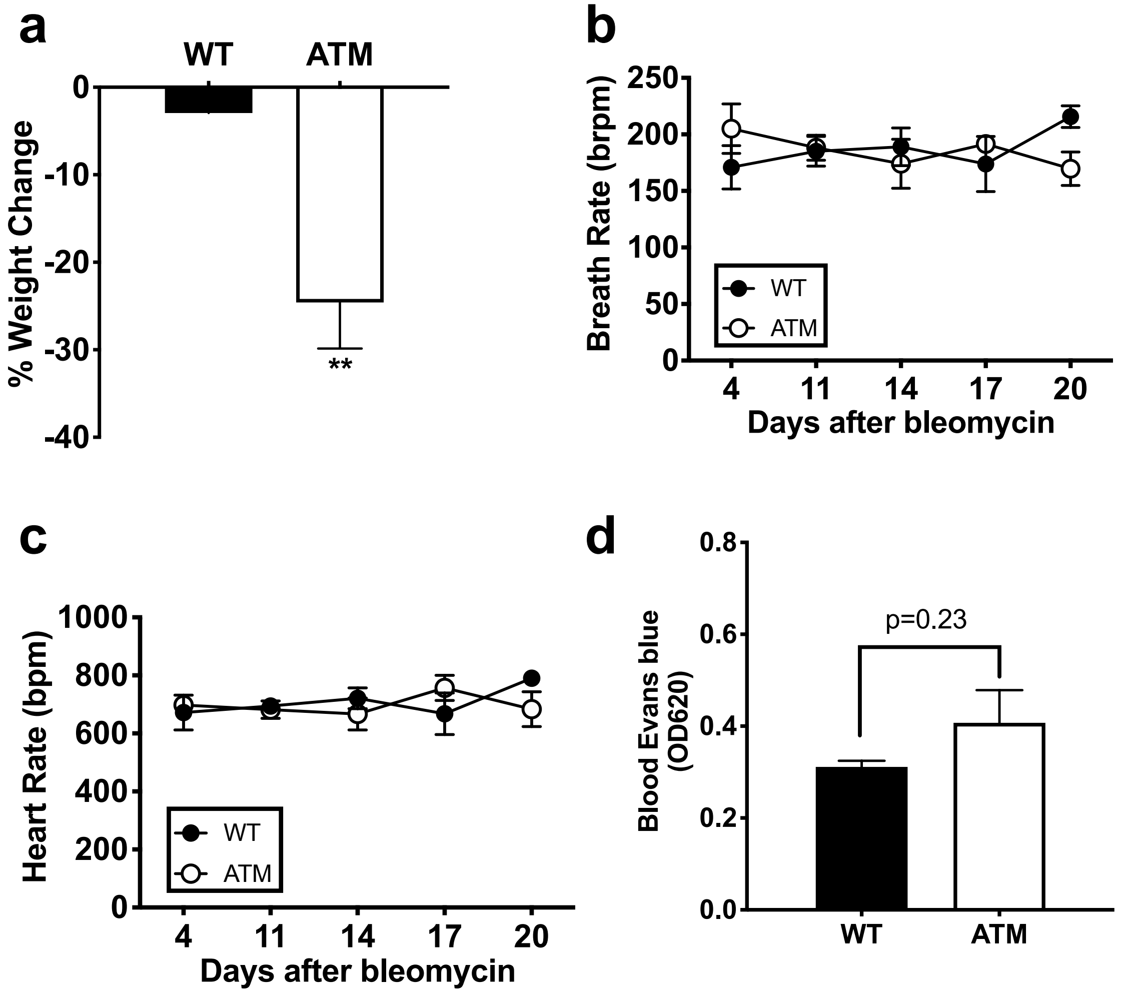

Supplementary Figure 2

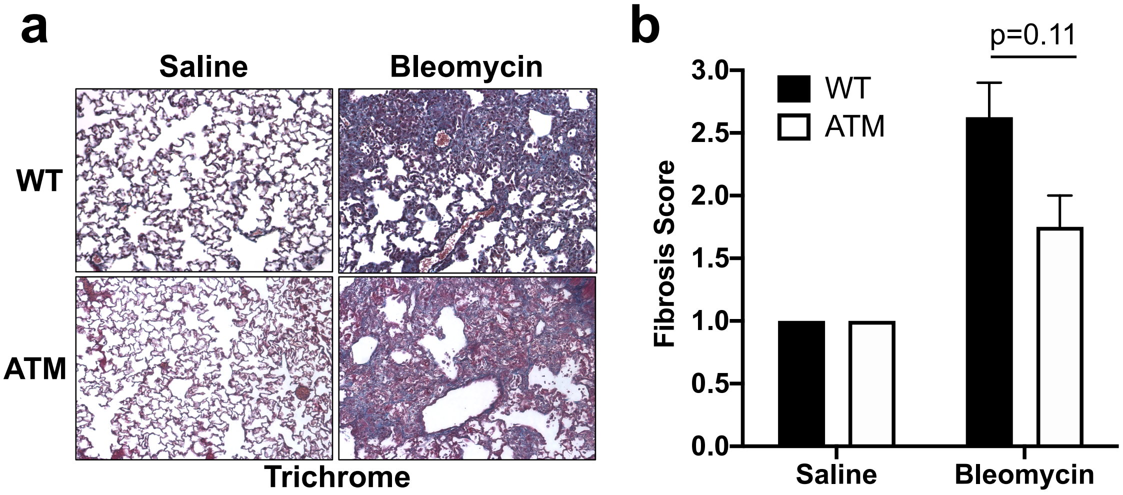

Supplementary Figure 3

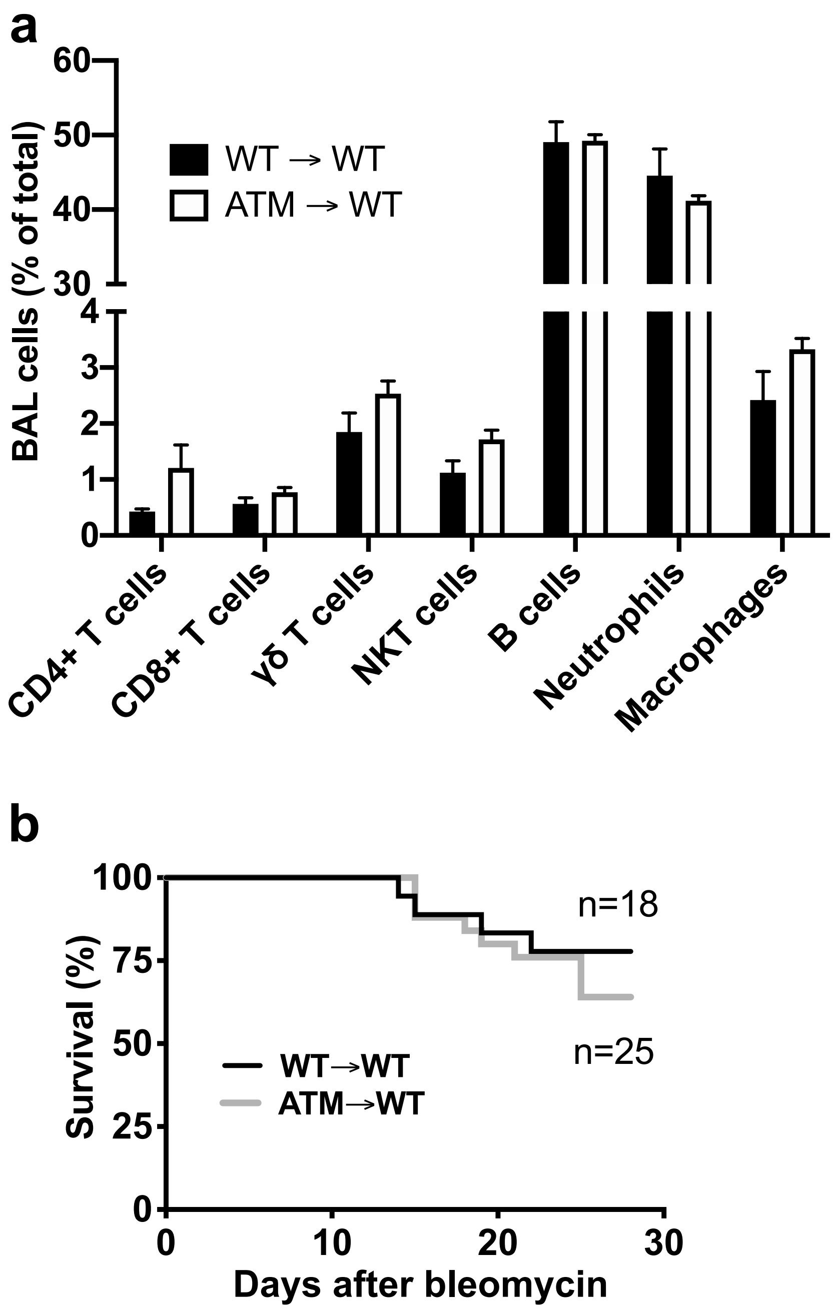

Supplement: Supplementary file 1 — Supplementary information. [file 41598_2021_83531_MOESM1_ESM.pdf]
